# Understanding experiences of potential harm among MSM (cis and trans) using HIV self-testing in the SELPHI randomised controlled trial in England and Wales: a mixed-methods study

**DOI:** 10.1136/sextrans-2023-055840

**Published:** 2023-08-22

**Authors:** T Charles Witzel, Emily Jay Nicholls, Leanne McCabe, Peter Weatherburn, Sheena McCormack, Christopher Bonell, Mitzy Gafos, Fiona C Lampe, Andrew Speakman, David Dunn, Denise Ward, Andrew N Phillips, Roger Pebody, Michelle M Gabriel, Yolanda Collaco-Moraes, Alison J Rodger, Fiona M Burns

**Affiliations:** 1 Department of Public Health, Environments and Society, London School of Hygiene & Tropical Medicine, London, UK; 2 Institute for Global Health, University College London, London, UK; 3 Medical Research Council Clinical Trials Unit, University College London, London, UK; 4 Social & Environmental Health Research, London School of Hygiene & Tropical Medicine, London, UK; 5 Global Health and Development, London School of Hygiene & Tropical Medicine, London, UK; 6 NAM, London, UK

**Keywords:** HIV, Diagnostic Screening Programs, Homosexuality, Male

## Abstract

**Background:**

The potential of HIV self-testing (HIVST) to cause harm is a concern hindering widespread implementation. The aim of this paper is to understand the relationship between HIVST and harm in SELPHI (An HIV Self-testing Public Health Intervention), the largest randomised trial of HIVST in a high-income country to date.

**Methods:**

10 111 cis and trans men who have sex with men (MSM) recruited online (geolocation social/sexual networking apps, social media), aged 16+, reporting previous anal intercourse and resident in England or Wales were first randomised 60/40 to baseline HIVST (baseline testing, BT) or not (no baseline testing, nBT) (randomisation A). BT participants reporting negative baseline test, sexual risk at 3 months and interest in further HIVST were randomised to three-monthly HIVST (repeat testing, RT) or not (no repeat testing, nRT) (randomisation B). All received an exit survey collecting data on harms (to relationships, well-being, false results or being pressured/persuaded to test). Nine participants reporting harm were interviewed in-depth about their experiences in an exploratory substudy; qualitative data were analysed narratively.

**Results:**

Baseline: predominantly cis MSM, 90% white, 88% gay, 47% university educated and 7% current/former pre-exposure prophylaxis (PrEP) users. Final survey response rate was: nBT=26% (1056/4062), BT=45% (1674/3741), nRT=41% (471/1147), RT=50% (581/1161).

Harms were rare and reported by 4% (n=138/3691) in exit surveys, with an additional two false positive results captured in other study surveys. 1% reported harm to relationships and to well-being in BT, nRT and RT combined. In all arms combined, being pressured or persuaded to test was reported by 1% (n=54/3678) and false positive results in 0.7% (n=34/4665).

Qualitative analysis revealed harms arose from the kit itself (technological harms), the intervention (intervention harms) or from the social context of the participant (socially emergent harms). Intervention and socially emergent harms did not reduce HIVST acceptability, whereas technological harms did.

**Discussion:**

HIVST harms were rare but strategies to link individuals experiencing harms with psychosocial support should be considered for HIVST scale-up.

**Trial registration number:**

ISRCTN20312003.

**WHAT IS ALREADY KNOWN ON THIS TOPIC**

- Increasing rates of ever and repeat testing is critical to supporting sustained reductions in HIV incidence for men who have sex with men (MSM; cis and transgender); HIV self-testing (HIVST) is a relatively novel approach which may support this key goal. Evidence about harms from HIVST among key populations is scant: a recent systematic review and meta-analysis of 10 randomised controlled trials comparing HIVST to standard testing among key populations found evidence of harms only in trials including female sex workers and none for MSM or trans people^5^. Observational evidence on HIVST implementation largely reports interpersonal conflict when HIVST is delivered by peers or partners.

**WHAT THIS STUDY ADDS**

- We demonstrate that harms from HIVST are very rare. When these do occur, harms can arise from the kit itself, from how the intervention functions or from the broader social circumstances of the end user which we term technological, intervention and socially emergent harms, respectively.

**HOW THIS STUDY MIGHT AFFECT RESEARCH, PRACTICE OR POLICY**

- This research will be encouraging to policy makers and commissioners reluctant to implement HIVST because of concerns about the potential for harm.

## Background

HIV self-testing (HIVST) involves a person collecting their own sample, processing their test and interpreting their result.^1 2^ A novel intervention, HIVST has advantages in reaching marginalised populations most affected by HIV, such as cis and trans men who have sex with men (MSM), by reducing testing barriers (eg, inaccessible clinics, stigma, opportunity cost).^2–4^ HIVST implementation has been hindered by concern about harms or adverse events in people offered HIVST, and which would not necessarily have occurred with clinic-based testing. We do not strictly define harm or adverse events in this paper as they have overlapping meanings, but we use the term harm as this is the norm in the wider HIVST literature.^5–7^

In the UK, policy makers and commissioners have been reluctant to endorse HIVST which was banned in 1992 because of concerns over test quality and the potential for self-harm in the absence of pre-and-post-test counselling and effective HIV treatment.^1 8^ These concerns persisted despite advances in treatment leading to normal life expectancies for people with diagnosed HIV.^1 9^ Further concerns focus on the potential of HIVST for coercive testing and the negative impacts of reduced support.^1 6^

Since legalisation in 2014, HIVST has been provided sporadically across the UK through pilot and demonstration projects and through our online randomised controlled trial (RCT) in England and Wales.^10–13^ SELPHI (An HIV Self-testing Public Health Intervention) recruited 10 111 men (cis and trans) who reported lifetime anal sex with men.

SELPHI provided the BioSure HIV self-test and had two randomisations. Randomisation A assessed whether provision of one free HIVST could increase confirmed diagnoses of prevalent infections, and found no significant difference between HIVST and standard of care.^14^ Randomisation B assessed whether the offer of repeat free HIVST could increase diagnoses of incident infections, and also found no difference between arms.^15^ Secondary outcomes included dramatically increased HIV testing uptake and frequency, without reductions in sexually transmitted infection testing.^14–16^

Despite significant concern from policy makers and in values and preferences research (conducted primarily with people who had not used HIVST), evidence about actual harms from HIVST use is scant. Research has focused on social harms (eg, coercion, intimate partner violence (IPV)) rather than harms from tests themselves (eg, false positives/negatives).^2 5 6^

A recent meta-analysis investigating HIVST outcomes in key populations found no reports of harm in seven RCTs recruiting MSM and trans people, and very few in three RCTs including female sex workers.^5^ However, it is not clear if harms data were routinely reported; one included RCT has since reported 8.5% of MSM (n=60/709) reported pressuring someone else to test, and 2.1% (n=15/715) being pressured themselves.^17^ Some evidence from observational studies exists in high-income settings, mostly confrontations with sexual partners when delivering HIVST.^18 19^

As the biggest HIVST RCT in a high-income setting and the largest including MSM to date, SELPHI provides a unique opportunity to investigate and characterise harms to guide HIVST implementation.

The aim of this study is to understand the relationship between HIVST and harm in SELPHI by exploring specific types of harm (relationship, well-being, pressure/persuasion to test and false positive/negative results), and the experiences of individuals who reported harm.

## Methods

This mixed-methods study uses SELPHI RCT data alongside an embedded qualitative substudy including participants who reported harm. We use an approach termed ‘following the thread’ whereby quantitative data are explored further through qualitative inquiry.^20^

### Trial design and measures

**Figure 1:**
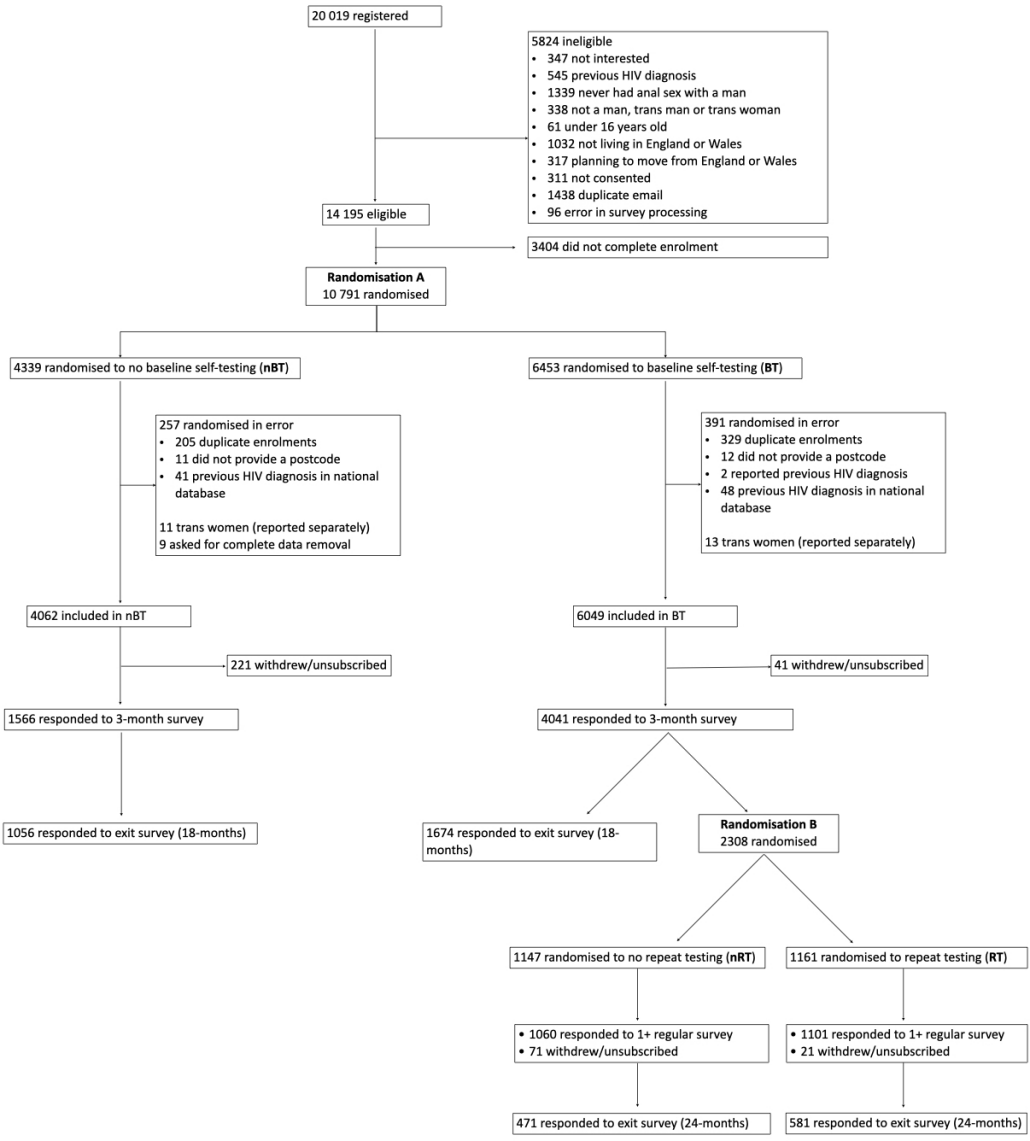
Trial Consolidated Standards of Reporting Trials (CONSORT) flow diagram.

SELPHI recruited MSM (cis/trans) and trans women with self-reported HIV negative/unknown status, who reported lifetime anal sex with men, through sexual networking apps and social media (February 2017 to March 2018). Data for all trans participants are reported separately.^13^ Trial protocol^11^ and intervention descriptions are also reported elsewhere.^10 12^ SELPHI was prospectively registered with the ISRCTN. Figure 1 provides a flow diagram with retention.

A total of 10 111 MSM completed baseline questionnaires, which included an HIV risk assessment, and were individually computer randomised 60/40 to receive one baseline HIVST (baseline testing, BT) versus no baseline HIVST (no baseline testing, nBT) (randomisation A). Participants in BT received a survey at 2 weeks and at 3 months, in nBT participants received a survey at 3 months. A final exit survey (18 months after enrolment) asked whether using HIVST or being in SELPHI led to negative impacts on relationships (family, partners, friends, coworkers), or negative impacts on well-being, and whether since joining SELPHI participants had been pressured or persuaded to HIV test when they did not want to.

Randomisation B occurred 3 months after randomisation A. Participants in BT who reported using the baseline HIVST, condomless anal sex with ≥1 male partner in previous 3 months and interest in further HIVST were randomised 50/50 to repeat three-monthly HIVST (repeat testing, RT) versus no repeat testing (nRT). RT participants received a survey 2 weeks after each HIVST delivery. nRT and RT participants received a regular survey every three months including a risk assessment. An exit survey (24 months after enrolment) included questions about harms.

In intervention arms (BT, nRT and RT), false positive results could be reported on the 2-week, 3-month and regular surveys (in RT). All participants were asked about false positive results from self-tests (provided by SELPHI and sourced elsewhere) at end of study surveys acknowledging that HIVST was available sporadically through pilot projects during follow-up and commercially at cost throughout.

### RCT analysis

Our complete case intention-to-treat analysis was not determined prior to trial implementation. We analysed harm types (relationships, well-being, pressure/persuasion to test) based on trial arm (nBT, BT, nRT, RT). Responses to questions regarding relationship harm, well-being harm and pressure to test were tabulated over numbers who responded to each question by trial arm. Participants reporting false positive results in 2-week, 3-month, regular and exit surveys were tabulated over all who reported an HIVST result or using HIVST during trial follow-up (including kits not sourced from SELPHI). To avoid double counting, participants from BT subsequently randomised a second time contributed only to nRT and RT endpoints. Stata V.16.1 was used for analysis.

### Qualitative study

We conducted an exploratory qualitative substudy examining participant accounts. A topic guide (online supplemental file 1) drew on themes emerging from a previous SELPHI sub-study^10^ and existing HIVST literature. This covered HIV testing experiences, engagement with SELPHI and the type(s) of harm reported.

10.1136/sextrans-2023-055840.supp1 Supplementary data

Participants who consented to contact for qualitative research and reported at least one harm type were invited to interviews. One experiencing a false negative interviewed in a different SELPHI study^21^ was included post hoc. Participants were screened during interview for harms not reported in surveys.

Interviews (June to August 2020) were conducted over Zoom and by telephone (because of COVID-19 restrictions) by the first and second authors, audio recorded and transcribed verbatim. Participants were given £30.

Analysis (conducted using QSR NVivo V.12) followed a narrative approach. Participant accounts were treated as self-contained stories, and elements coded based on position within the narrative (eg, initial explanation, contributing features, critical point, resolution). Results were assessed for clarity/coherence by TCW, EJN, PW, AR and FB. As this study was primarily exploratory, we did not attempt to assess saturation.

This study involves human participants and was approved by the University College London (ref: 9233/001) and the London School of Hygiene & Tropical Medicine (ref: 17985) research ethics committees. Participants gave informed consent to participate in the study before taking part.

## Results

SELPHI recruited 10 111 MSM (cis/trans); predominantly cis MSM, 90% white, 88% gay, 47% university educated and 7% current or former pre-exposure prophylaxis users. Baseline details are available elsewhere.^13^ Exit surveys were completed by 26–50% of participants: nBT 26% (1056/4062), BT 45% (1674/3741), nRT 41% (471/1147) and RT 50% (581/1161).

### Prevalence and distribution of harms

Harms were reported by 4% (n=138/3691) of those who responded to one or more harm question on the exit survey. Of those reporting harm, 9% (n=13/138) of participants reported >1 harm and 1% (n=2/138) >2 harms. Additionally, 0.7% (n=2/3061) reported a false positive elsewhere in the trial.

Negative impacts to relationships from participating in SELPHI or using HIVST kits were reported by 1% (n=25/2675) in BT, nRT and RT combined, with no substantial differences by trial arm (table 1). Impacts were on partners (n=10), other people (n=7), family (n=5), friendships (n=5) and work (n=3). Negative impacts on family were primarily relationship breakdowns (n=5); negative impacts on partner relationships were primarily arguments (n=5).

**Table 1:** Types of harm by trial arm

Type of harm	No baseline testing (nBT) % (n/N)	Baseline testing (BT) % (n/N)	No repeat testing (nRT) % (n/N)	Repeat testing (RT) % (n/N)	Overall % (n/N)
Relationships	N/A	1 (15/1626)	0.2 (1/468)	2 (9/581)	1% (25/2675)*
Well-being	N/A	1 (18/1611)	2 (8/467)	2 (11/580)	1% (37/2658)*
Pressured/persuaded to test	1 (14/1013)	1 (21/1615)	2 (8/471)	2 (11/579)	1% (54/3678)
False positive	0.8 (3/359)	0.5 (9/2013)	1.0 (11/1136)	1.0 (11/1157)	0.7% (34/4665)

*Data not collected for nBT.

**Table 2:** Qualitative study participant demographics

Demographic variable	n
Age	
18–25	3
26–35	3
36–45	1
45+	2
Sexual orientation	
Gay	8
Undisclosed	1
Testing history at enrolment	
Never	1
<12 months	5
>12 months	3

Ethnicity not reported because of risk of deductive disclosure.

Negative impacts on well-being were reported by 1% (n=37/2658) in BT, nRT and RT combined. Being pressured or persuaded to test when a participant did not want to was reported by 1% (n=54/3678). False positive test results were reported by 0.7% (n=34/4665) of participants overall, and similarly across trial arms. Two were reported during the trial, and a further 32 in end of study surveys. No participant reported multiple false positive results (table 1).

### Experiences of harms

Ninety-seven who reported harm also provided consent for further contact; 78 were contacted and nine completed an interview. Table 2 provides participant demographics.

Qualitative analysis (see online supplemental file 2 for expanded version) revealed three categories of harms experienced during SELPHI: caused by the HIVST itself (technological harms), caused by the intervention more broadly (intervention harms) or arising from interactions between HIVST/the intervention and the social circumstances of the individual (socially emergent harms).

10.1136/sextrans-2023-055840.supp2 Supplementary data

#### Technological harms

Three participants reporting harms due to the technology itself were interviewed, two had a false positive and one a false negative result. One of the false positive reports, however, was from a non-trial HIV self-sampling (HIVSS) kit (an HIV test where a person takes a sample and sends it to a laboratory for processing) rather than an HIVST kit and their data were excluded.

Both remaining participants’ experience of harm emerged from the test itself and did not have external influences exacerbating the outcome. The participant who reported the false positive HIVST described a difficult series of emotions when reading the result, including guilt and shame. He sought support from his wider social network and attended a clinic for confirmatory testing within 24 hours where support was provided. A rapid test was not conducted in clinic, and the participant waited several days for a result, which was negative. The false positive HIVST undermined the participants’ well-being, leading to the termination of a fledgling relationship.

At the time […] …I wanted to be on my own for three months and get the next result. […] I pushed a lot of people away. I didn’t really want to be with anybody or see anybody or be in a relationship. So, I would just keep away [from a man he was dating]. Very much felt isolated for three months until I could get another result which I was happy with. […] After the negative one I wanted a second one just to confirm that. It did affect relationships, like I didn’t really want to be sexual at that time. (Cis man aged 26–35 years. Baseline testing, false positive result)

The false negative result had a less clear impact: this man had a negative result from an HIVST, several days later a health condition led to his general practitioner testing him for HIV again, this result was positive. The participant felt angry and frustrated, he attempted to access support via the SELPHI website and a helpline run by a voluntary sector organisation. He found it challenging to access information and the offered support did not meet his needs.

I didn’t find it helpful. I just found it like, I suppose it was like a mental health study. So, [HIV support helpline] was, I think it was offering counselling but I didn’t feel I needed counselling. I only felt I needed someone to talk to, not a counsellor. (Cis man aged 26–35 years. Baseline testing, false negative result)

Both participants reported low HIVST acceptability following their experiences, were suspicious of the technology and reluctant to endorse self-testing.

#### Intervention harms

Harms related to intervention function were reported by two individuals. Accounts focused on feelings of guilt and shame when they completed online risk surveys.

Once you’ve entered into the trial and then it’s like, so, why did you feel the need [to test]? What sort of person do you think you have become where you feel that you have to be tested? And it also made me think, why do I feel the need to be tested so regularly at the normal clinic? […] Am I living a normal sort of lifestyle? Is my lifestyle, is it out of control? Is it the sort of lifestyle that I want? And how, at my age, did I get myself into this position? (Cis man aged 45+ years. Repeat testing, well-being harm)

For both, narratives describing their experiences focused on their internal monologue and their circumstances. For one, this was living on his own, and for the other it was related to not being open about his sexual orientation with his family. These narratives were focused on guilt, shame and loss of control. Although the surveys and the cyclical nature of the intervention triggered these feelings, using HIVST kits in the home increased this because of the incursion of healthcare into the private sphere, an issue specific to HIVST:

I guess because it’s quite a clinical thing actually, you know, when you think about it. It’s quite a clinical thing to be doing in your own room. It’s like, you know, something you would ordinarily have done by someone who’s trained, but you’re having it in a different way and you’re having it in a completely different setting. So maybe it would be easier to, kind of, make it be I guess more normal in that clinical [setting]… (Cis man aged 18–25 years. Repeat testing, well-being harm)

#### Socially emergent harms

Four participants reported harms arising from their social contexts. For one, this was a negative impact on a relationship, for three this was pressure to test for HIV when they did not want to.

One participant reported a difficult relationship with his partner which was characterised by jealousy and suspicion. His partner’s discovery of his HIVST led to discord and contributed to eventual relationship breakdown. His narrative, however, described the inevitability of the outcome: had the discovery of the kit not provoked breakdown another event would have precipitated it.

Pressure or persuasion to test during SELPHI was experienced by three individuals in different circumstances: for one, it came from a friend concerned for his health. For another, pressure came from a partner in response to his own worsening mental health due to HIV anxiety. The final participant described being forced to test in a clinic by the police after being violently sexually assaulted when he would have preferred to use HIVST; his experience was thus not related to HIVST or SELPHI participation.

Both individuals pressured to test by those in social networks described ambivalence and anxiety around testing for HIV while also recognising their own unmet testing need. For one, this need was the source of significant stress and negatively impacted their well-being. Although both described significant ambivalence around the experience, they were happy they had tested in retrospect and felt more confident with future testing:

It [testing] just seems so much more reachable. I feel comfortable doing it now. And I think, more than anything, it’s put my mind at rest that it is getting easier to be able to be tested for this. (Cis man aged 18–25 years. Baseline testing, pressured to test)

For participants pressured to test when they did not want to, HIVST through SELPHI was simply the most accessible test available to them at that time. If HIVST was not available this pressure likely would have led them to test using another modality.

## Discussion

In this analysis, harm related to HIVST was extremely rare and experienced by 4% who completed exit surveys. Exploratory qualitative data suggest HIVST harms arise either from the technology itself, are generated by the intervention more broadly or emerge from individuals’ specific social circumstances. Harm could not always be attributed solely to HIVST, in some instances HIVST was merely a catalyst.

It is inevitable due to technology limitations that some will experience false positive and false negative HIVST results, similar to other testing options.^1 22–24^ Supportive information for those who receive a positive result from a self-test should include seeking timely confirmatory testing. False negatives, which are rarer, given imperatives to develop tests with high sensitivity, are more challenging to address; the most practical solution is to encourage repeat testing and to prioritise HIVSTs with high sensitivity. Indeed, the participant reporting the false negative may have tested within the window period following HIV exposure. The BioSure test has a longer window period than clinic-based fourth-generation HIV antigen/antibody tests. Nevertheless, the participant understood his result as a false negative, and providers should consider the emotional and public health implications. This underlines the importance of providing clear information on test sensitivity, specificity and window period to enable individuals to correctly interpret their results in the context of test capabilities.

Harms emerging from intervention components resulted from the intervention functioning as theorised, but with a more extreme outcome than anticipated. The goal of risk assessment was to provide a reflective experience on sexual risks.^10 12^ For a very small number, this exacerbated self-stigma/internalised homophobia, negatively impacting on well-being. This is likely to be an issue for MSM and other key populations who face marginalisation and exclusion in society. Socially emergent harms pose a similar challenge; they are difficult to predict, are interpersonal, largely independent of the HIVST/intervention and therefore cannot reasonably be anticipated. Packaging HIVST with broader supportive resources, including clear links to relationship and other psychosocial support, is pragmatic given their rarity.

Being pressured or persuaded to test during SELPHI was not always related to HIVST. Although unwanted pressure to test is likely to occur with other modalities, because self-testing is convenient and removed from clinical spaces, it may well lead to increases in acquiescence to testing pressure. For both individuals pressured to use an HIVST, if self-testing had not been available they likely would have used another modality. Further, both were ultimately happy they tested and, in line with other research from China, reported positive attitudes towards HIVST. Understanding pressure/persuasion in context is therefore important.^25^

Our findings are novel and, to our knowledge, the most in-depth from a high-income setting exploring HIVST harms.^18 19^ Our results correspond with research from Southern Africa demonstrating potential harms were most likely to emerge within relationships,^26^ or be related to unwanted pressure to test.^27^

Given HIVST’s substantial benefits in providing MSM with an empowering testing option which increases testing uptake and diagnosis,^5 28^ these rare harms should not pose a barrier to implementation. However, future programming should monitor harms across the three domains identified: technological, intervention and socially emergent harms.

### Limitations

This study has important limitations. Completion rates for the final surveys were low, especially in nBT, the arm in which participants received no HIVST. This means that harm frequency may be over-estimated if those experiencing harm were more likely to complete exit surveys, or under-reported if they were less likely to do so.

There is sometimes confusion between HIVST and HIVSS. Given that one participant mistakenly reported a false positive as being from an HIVST but which was actually from HIVSS, which has a comparatively high false positive rate,^29^ the number of false positives reported in this study are likely upper estimates due to confusion between technologies.

We did not systematically collect SELPHI data on IPV. Although none of the participants interviewed reported this, it may have been experienced by others during the trial.^30^

Finally given the relatively small sample size in the qualitative component due to the small number of harms and because not all who consented to follow-up agreed to be interviewed, these data are exploratory rather than indicative of all experiences of harms.

## Conclusions

Harms were extremely rare, reported by 4% of participants. Qualitative analysis reveals harms emerge from the technology, from intervention function or from individuals’ social/interpersonal circumstances; these were not always directly attributable to HIVST. Strategies managing harms should focus on providing links to psychosocial support. Given the rarity of harms and the wider benefits of HIVST, this should not be a barrier to implementation.

## Data Availability

Data are available upon reasonable request.

## References

[1] Witzel TC , Rodger AJ . New initiatives to develop self-testing for HIV. Curr Opin Infect Dis 2017;30:50–7. 10.1097/QCO.0000000000000336 27849635

[2] Figueroa C , Johnson C , Verster A , et al . Attitudes and acceptability on HIV self-testing among key populations: a literature review. AIDS Behav 2015;19:1949–65. 10.1007/s10461-015-1097-8 26054390 PMC4598350

[3] Pant Pai N , Sharma J , Shivkumar S , et al . Supervised and unsupervised self-testing for HIV in high- and low-risk populations: a systematic review. PLoS Med 2013;10:e1001414. 10.1371/journal.pmed.1001414 23565066 PMC3614510

[4] Witzel TC , Rodger AJ , Burns FM , et al . HIV self-testing among men who have sex with men (MSM) in the UK: a qualitative study of barriers and facilitators. PLoS One 2016;11:e0162713. 10.1371/journal.pone.0162713 27611777 PMC5017738

[5] Witzel TC , Eshun-Wilson I , Jamil MS , et al . Comparing the effects of HIV self-testing to standard HIV testing for key populations: a systematic review and meta-analysis. BMC Med 2020;18:381. 10.1186/s12916-020-01835-z 33267890 PMC7713313

[6] Brown AN , Djimeu EW , Cameron DB . A review of the evidence of harm from self-tests. AIDS Behav 2014;18 Suppl 4:S445–9. 10.1007/s10461-014-0831-y 24989129 PMC4094790

[7] Jamil MS , Eshun-Wilson I , Witzel TC , et al . Examining the effects of HIV self-testing compared to standard HIV testing services in the general population: a systematic review and meta-analysis. EClinicalMedicine 2021;38:100991. 10.1016/j.eclinm.2021.100991 34278282 PMC8271120

[8] Myers JE , El-Sadr WM , Zerbe A , et al . Rapid HIV self-testing: long in coming but opportunities beckon. AIDS 2013;27:1687–95. 10.1097/QAD.0b013e32835fd7a0 23807269

[9] Teeraananchai S , Kerr SJ , Amin J , et al . Life expectancy of HIV-positive people after starting combination antiretroviral therapy: a meta-analysis. HIV Med 2017;18:256–66. 10.1111/hiv.12421 27578404

[10] Witzel TC , Bourne A , Burns FM , et al . HIV self-testing intervention experiences and kit usability: results from a qualitative study among men who have sex with men in the SELPHI (Self-Testing Public Health Intervention) randomized controlled trial in England and Wales. HIV Med 2019. 10.1111/hiv.12818 PMC7065141 31821698

[11] Gabriel MM , Dunn DT , Speakman A , et al . Protocol, rationale and design of SELPHI: a randomised controlled trial assessing whether offering free HIV self-testing kits via the Internet increases the rate of HIV diagnosis. BMC Infect Dis 2018;18:531. 10.1186/s12879-018-3433-x 30352556 PMC6199717

[12] Witzel TC , Weatherburn P , Bourne A , et al . Exploring mechanisms of action: using a testing typology to understand intervention performance in an HIV self-testing RCT in England and Wales. Int J Environ Res Public Health 2020;17:466. 10.3390/ijerph17020466 31936798 PMC7014239

[13] Rodger AJ , Dunn D , McCabe L , et al . Sexual risk and HIV testing disconnect in men who have sex with men (MSM) recruited to an online HIV self‐testing trial. HIV Med 2020;21:588–98. 10.1111/hiv.12919 32776431

[14] Rodger AJ , McCabe L , Phillips AN , et al . Free HIV self-test for identification and linkage to care of previously undetected HIV infection in men who have sex with men in England and Wales (SELPHI): an open-label, Internet-based, randomised controlled trial. Lancet HIV 2022;9:e838–47. 10.1016/S2352-3018(22)00266-1 36460023 PMC7614584

[15] McCabe L , Rodger A , Phillips A , et al ., eds. Can the offer of regular HIV self-testing kits reduce time to HIV diagnosis in MSM? Results from the SELPHI RCT. In: Journal of the International Aids Society. Southern Gate, Chichester PO19 8SQ, W…: John Wiley & Sons Ltd The Atrium, 2021.

[16] Witzel TC , Wright T , McCabe L , et al . Impact and acceptability of HIV self-testing for trans men and trans women: a mixed-methods subgroup analysis of the SELPHI randomised controlled trial and process evaluation in England and Wales. EClinicalMedicine 2021;32:100700. 10.1016/j.eclinm.2020.100700 33681732 PMC7910695

[17] Gwynn M , Chavez PR , Borkowf CB , et al . Pressure to use HIV self-tests among men who have sex with men, United States, 2015–2016. AIDS Behav 2022;26:623–30. 10.1007/s10461-021-03422-6 34406551

[18] Giguere R , Lopez-Rios J , Frasca T , et al . Use of HIV self-testing kits to screen clients among transgender female sex workers in New York and Puerto Rico. AIDS Behav 2020;24:506–15. 10.1007/s10461-019-02730-2 31865516 PMC7187402

[19] Balán IC , Carballo-Diéguez A , Frasca T , et al . The impact of rapid HIV home test use with sexual partners on subsequent sexual behavior among men who have sex with men. AIDS Behav 2014;18:254–62. 10.1007/s10461-013-0497-x 23657758 PMC3815512

[20] O’Cathain A , Murphy E , Nicholl J . Three techniques for integrating data in mixed methods studies. BMJ 2010;341:bmj.c4587. 10.1136/bmj.c4587 20851841

[21] Nicholls EJ , Samba P , McCabe L , et al . Experiences of and attitudes towards HIV testing for Asian, black and Latin American men who have sex with men (MSM) in the SELPHI (HIV self-testing public health intervention) randomized controlled trial in England and Wales: implications for HIV self-testing. BMC Public Health 2022;22:809. 10.1186/s12889-022-13189-7 35459233 PMC9034480

[22] Pai NP , Balram B , Shivkumar S , et al . Head-to-head comparison of accuracy of a rapid point-of-care HIV test with oral versus whole-blood specimens: a systematic review and meta-analysis. Lancet Infect Dis 2012;12:373–80. 10.1016/S1473-3099(11)70368-1 22277215

[23] Stevens DR , Vrana CJ , Dlin RE , et al . A global review of HIV self-testing: themes and implications. AIDS Behav 2018;22:497–512. 10.1007/s10461-017-1707-8 28155039 PMC5910655

[24] Figueroa C , Johnson C , Ford N , et al . Reliability of HIV rapid diagnostic tests for self-testing compared with testing by health-care workers: a systematic review and meta-analysis. Lancet HIV 2018;5:e277–90. 10.1016/S2352-3018(18)30044-4 29703707 PMC5986793

[25] Ong JJ , Wu D , Huang W , et al . Pressured HIV testing "in the name of love": a mixed methods analysis of pressured HIV testing among men who have sex with men in China. J Int AIDS Soc 2018;21:e25098. 10.1002/jia2.25098 29577598 PMC5867275

[26] Kumwenda MK , Johnson CC , Choko AT , et al . Exploring social harms during distribution of HIV self-testing kits using mixed-methods approaches in Malawi. J Int AIDS Soc 2019;22 Suppl 1:e25251. 10.1002/jia2.25251 30907508 PMC6432111

[27] Lora W , Chipeta E , Desmond N . Understanding coercion in the context of semi-supervised HIV self-testing in urban Blantyre. AIDS 2016: 21st International AIDS Conference; 18-22nd July 2016; Durban, South Africa, 2016

[28] Eshun-Wilson I , Jamil MS , Witzel TC , et al . A systematic review and network meta-analyses to assess the effectiveness of human immunodeficiency virus (HIV) self-testing distribution strategies. Clin Infect Dis 2021;73:e1018–28. 10.1093/cid/ciab029 34398952 PMC8366833

[29] Page M , Atabani SF , Wood M , et al . Dried blood spot and mini-tube blood sample collection kits for postal HIV testing services: a comparative review of successes in a real-world setting. Sex Transm Infect 2019;95:43–5. 10.1136/sextrans-2018-053567 30072393

[30] Miltz AR , Lampe FC , Bacchus LJ , et al . Intimate partner violence, depression, and sexual behaviour among gay, bisexual and other men who have sex with men in the PROUD trial. BMC Public Health 2019;19:431. 10.1186/s12889-019-6757-6 31023281 PMC6482482

