# Supplementary material for: Understanding experiences of potential harm among MSM (cis and trans) using HIV self-testing in the SELPHI randomised controlled trial in England and Wales: a mixed-methods study
Source: Sex Transm Infect. 2023 Aug 22;99(8):534–40. doi: 10.1136/sextrans-2023-055840 (PMC10715485; doi:10.1136/sextrans-2023-055840)
Supplement: Supplementary data [file sextrans-2023-055840supp001.pdf]

Supplementary file 1: topic guides

Topic guide 1: Social harms

1. Introductions

- Researcher, Sigma, LSHTM
- This is an interview to hear your thoughts on HIVST. You've been invited because of your involvement with the SELPHI study. I'll ask you some specific questions about yourself, and some questions about your history. Feel free to answer them however you see fit. There are no right or wrong answers etc.
- Highlight confidentiality

2. Ethics

- We would like to record the interview so we don't miss any of what you say.
- Explain how we will use and protect the data
- Do you have any questions?
- Are you happy to proceed?

3. HIV/STI testing history

- Before you joined SELPHI, had you ever tested for HIV?

If no

- Had you considered HIV testing before?
- What had stopped you?
- How about STI screening? What have your experiences of that been like?
- Can you tell me about the last time you heard something about HIV testing? From whom? Where? What was the message? Did this feel relevant to you?
- What did it make you think? Was this different to other messages/images about HIV testing that you've seen in the past? How?

If yes

- Can you tell me about the first time you tested for HIV?
- And did you also have an STI screen? Tell me about that experience.
- Can you remember what prompted you to seek testing the first time you went?
- What was the experience like? Where did you go? How did you choose to test that way?
- What kind of support were you provided with? Was this what you wanted?
- Can you think of anything else that would have been helpful for you in that situation?
- How about the last time you tested before SELPHI? Can you tell me a bit more about that?
- Where did you go? How did you make that decision? What kind of support did you get? Is this what you wanted at the time?
- Now tell me about the last time you tested before joining SELPHI? What was your motivation for testing?
- Where did you go? How did you make that decision? What support did you get? Is this what you wanted at the time?
- Did you have a testing pattern? What usually motivates you to test?

3. Coercion (For those who report pressure to test)

- *(if no coercion reported)* Since joining SELPHI has anyone pressured or persuaded you to test for HIV when you did not want to?
- *(if coercion reported)* You mentioned that since joining SELPHI someone has pressured or persuaded you to test for HIV when you did not want to. Was that using an HIVST from us or was it testing a different way?
- Can you tell me a bit about that experience?
- Who was the person? How did they try to persuade you?
- What was going through your mind at the time?
- How did you react? What concerns did you have at the time about their behaviour?
- What was the outcome? Did you test?
- How did you feel after this experience? Did you tell anyone?
- What kind of support did you get?
- What kind of support would have been helpful that you didn't have?
- How has this experience shaped how you think about testing?
- (Skip sections 4,5, 6 & 7) unless these harms were indicated on final survey)

4. Initial engagement with HIVST & SELPHI

- When did you first hear about HIVST?
- What were your initial thoughts?
- How did you hear about the SELPHI study? What make you decide to take part?
- How did you find the process of signing up? Was there anything difficult? Anything that you didn't understand?
- How long did it take for your kit to arrive?

5. Experience of HIVST & trial infrastructure

- **[Show kit with accompanying sleeve]** This is the same version of the test we sent you. Can you remember what your first impressions of it were? (Make sure sleeve and kit itself are covered in conversation)
- Thinking back to when you first took the self-test, what was going through your mind? Tell me everything you can about the first time you used HIVST (*Prompt*: Where were you? did you think there was a possibility of a positive result?).
- Did you decide to take the test by yourself or was there someone with you?
- Did you read the instructions? How many times?
  (*Prompt:* if not answered: What did you make of them? Did you watch any of the videos online? Did the kit look easy to use?)
  (*Prompt*: Do you think you made any mistakes? Was it difficult to use the lancet?)
- Describe your experience of reading the result.
- What did you do afterwards? Did you tell anyone? What kind of support did you look for?
- Did you trust the result that your test gave you?

6. Relationship discord

- *(if no relationship discord disclosed on final survey)* Have you had any relationship issues as a result of your participation in SELPHI?

- *(If relationship discord reported in final survey)* In the final survey you indicated that you had relationship issues with you (friend / partner / family member / workplace) because of your experiences in SELPHI.
- Can you tell me more about that?
- (*open ended discussion exploring what happened*)
- Were these underlying issues which were made worse or was this the first time something like this had happened?
- How did you deal with the situation? Was there somewhere you could get support?
- Was there any support which we could have provided through the trial which would have helped in your situation?
- How has this experience shaped the way you think about HIV testing?

7. Wellbeing issues

- *(if no wellbeing issues disclosed on final survey)* Have you had any wellbeing issues as a result of your participation in SELPHI?
- *(if wellbeing issues disclosed on final survey)* In the final survey you indicated that your wellbeing suffered as a result of your engagement with SELPHI.
- Can you tell me more about what happened?
- (*open ended discussion exploring the issues*)
- Were these underlying issues which were made worse or was this the first time something like this had happened?
- How did you deal with the situation? Was there somewhere you could get support?
- Was there any support which we could have provided through the trial which would have helped in your situation?
- How has this experience shaped the way you think about HIV testing?

8. After HIVST

- Have you talked to anyone about HIVST? Did you tell anyone you had taken a self-test? What did you tell them about the experience? What do you think they thought about it?
- Would you use HIVST again if it was available to you? If HIVST was free what difference would that make to your testing?
- Is there someone or a group of people you think HIVST is particularly good for? Anyone that it is not suitable for?

Topic guide 2: False positive results

1. Introductions

- Researcher, Sigma, LSHTM
- This is an interview to hear your thoughts on HIVST. You've been invited because of your involvement with the SELPHI study. I'll ask you some specific questions about yourself, and some questions about your history. Feel free to answer them however you see fit. There are no right or wrong answers etc.
- Highlight confidentiality nothing shared with GICs etc.

2. Ethics

- We would like to record the interview so we don't miss any of what you say.
- Explain how we will use and protect the data
- Do you have any questions?
- Are you happy to proceed?

3. HIV/STI testing history

- Before you joined SELPHI, had you ever tested for HIV?

If no

- Had you considered HIV testing before?
- What had stopped you?
- How about STI screening? What have your experiences of that been like?
- Can you tell me about the last time you heard something about HIV testing? From whom? Where? What was the message? Did this feel relevant to you?
- What did it make you think? Was this different to other messages/images about HIV testing that you've seen in the past? How?

If yes

- Can you tell me about the first time you tested for HIV?
- And did you also have an STI screen? Tell me about that experience.
- Can you remember what prompted you to seek testing the first time you went?
- What was the experience like? Where did you go? How did you choose to test that way?
- What kind of support were you provided with? Was this what you wanted?
- Can you think of anything else that would have been helpful for you in that situation?
- How about the last time you tested before SELPHI? Can you tell me a bit more about that?
- Where did you go? How did you make that decision? What kind of support did you get? Is this what you wanted at the time?

4. Initial engagement with HIVST & SELPHI

- When did you first hear about HIVST?
- What were your initial thoughts?
- How did you hear about the SELPHI study? What make you decide to take part?
- How did you find the process of signing up? Was there anything difficult? Anything that you didn't understand? [**Show registration and enrolment survey**]
- How long did it take for your kit to arrive?

5. Experience of HIVST & trial infrastructure

- **[Show kit with accompanying sleeve]** This is the same version of the test we sent you. Can you remember what your first impressions of it were? (Make sure sleeve and kit itself are covered in conversation)
- Thinking back to when you first took the self-test, what was going through your mind? Tell me everything you can about the first time you used HIVST (*Prompt*: Where were you? did you think there was a possibility of a positive result?).
- Did you decide to take the test by yourself or was there someone with you?

- Did you read the instructions? How many times?
  (*Prompt:* if not answered: What did you make of them? Did you watch any of the videos online? Did the kit look easy to use?)
  (*Prompt*: Do you think you made any mistakes? Was it difficult to use the lancet?)
- Describe your experience of reading the result.
- (*Prompt:* How did you feel when you saw the positive result?)
- What did you do afterwards? Did you tell anyone? What kind of support did you look for?
- Did you trust the result that your test gave you?
- What happened when you went to the clinic? How long did it take for you to have another test? What was the outcome of that?
- How did you feel about the support provided at the clinic?
- Was there any support which we could have provided with the kit which would have helped in your situation?
- Can you describe what impact, if any, this experience has had on your mental health or wellbeing?
- Do you remember receiving a follow-up survey about two weeks after you took the test? What were your impressions? [**Show email 1 and survey 2**]
- Do you remember receiving a follow-up survey about two weeks after you took the test? What were your impressions? [**Show email 2 and survey 3**]
- How has this experience shaped the way you think about HIV testing?

6. After HIVST

- Have you talked to anyone about HIVST? Did you tell anyone you had taken a self-test? What did you tell them about the experience? What do you think they thought about it?
- Would you use HIVST again if it was available to you?
- Is there someone or a group of people you think HIVST is particularly good for? Anyone that it is not suitable for?
