# Supplementary material for: Understanding experiences of potential harm among MSM (cis and trans) using HIV self-testing in the SELPHI randomised controlled trial in England and Wales: a mixed-methods study
Source: Sex Transm Infect. 2023 Aug 22;99(8):534–40. doi: 10.1136/sextrans-2023-055840 (PMC10715485; doi:10.1136/sextrans-2023-055840)
Supplement: Supplementary data [file sextrans-2023-055840supp002.pdf]

BMJ Publishing Group Limited (BMJ) disclaims all liability and responsibility arising from any reliance placed on this supplemental material which has been supplied by the author(s)

Experiences of harms

Qualitative analysis of 9 interviews revealed three categories of harms experienced during SELPHI: those caused by the HIVST itself (technological harms), those caused by the intervention more broadly (intervention harms) and those which arose from interactions between HIVST/the intervention and the social circumstances of the individual (socially emergent harms).

Technological harms

Three participants who reported harms due to the technology itself were interviewed, two a false positive and one a false negative result. During interview it was found that one of the false positive reports was from an HIV self-sampling (HIVSS) kit accessed outside of the trial (an HIV test where a person takes a sample and sends it to a lab for processing) rather than an HIVST kit; their data were excluded.

Both remaining participants' experience of harm emerged from the test itself and did not have external influences exacerbating the outcome. The participant who reported the false positive HIVST result described a difficult series of emotions when reading the result, including guilt and shame. He sought support from his wider social network and attended a clinic for confirmatory testing within 24-hours where emotional support was provided.

> *She* [the nurse] *was very good. She gave me some advice before taking the test. There was a lot of support if it was to come back positive.* […] *but I remember saying, you know, is there a chance that this could come back…could the SELPHI test be wrong* […] *I think she felt like it's probably going to be positive. Then I was like oh my. So that probably made me a little bit more anxious as well. I think in my mind I was still hoping that someone would tell me that the test was wrong*. (2~~6~~5-3~~5~~4 year-old cis-man. Baseline testing, false positive result)

A rapid test was not conducted in clinic, and the participant waited several days for a result, which was negative. The false positive HIVST undermined the participants well-being, leading to the termination of a fledgling relationship.

> *At the time* […] *…I wanted to be on my own for three months and get the next result.* […] *I pushed a lot of people away. I didn't really want to be with anybody or see anybody or be in a relationship. So, I would just keep away* [from a man he was dating]. *Very much felt isolated for three months until I could get another result which I was happy with.* […] *After the negative one I wanted a second one just to confirm that. It did affect relationships, like I didn't really want to be sexual at that time.* (2~~6~~5-3~~5~~4 year-old cis-man. Baseline testing, false positive result).

The false negative result had a less clear impact: this man had a negative result from an HIVST, several days later a health condition led to his GP testing him for HIV again, this result was positive.

> *However, I went to* [redacted] *in* [redacted] *and a doctor there tested me because I was having some complications at the time*, [redacted] *and the doctor asked me if he could do an HIV test. I said to him, 'I've just done one a few days ago and it came back negative.' He said, 'okay I just want to check anyway.'* […] *So he drew some blood from me, and about three days later he came back and said I was HIV positive. I said to him, 'how can that be, I just did the test.' He asked me how I did the test. I said to him* [that] *I got a test from Selfie and when I did the test it came back negative. So the doctor said to me, self-testing is not very accurate and he would not advise it.* (~~25~~26-35~~4~~ year-old cis-man. Baseline testing, false negative)

The participant felt angry and frustrated, he attempted to access support via the SELPHI website and a helpline run by a voluntary sector organisation. He found information was challenging to access and the support being offered was not what he required.

> *I didn't find it helpful. I just found it like, I suppose it was like a mental health study. So,* [HIV support helpline] *was, I think it was offering counselling but I didn't feel I needed counselling. I only felt I needed someone to talk to, not a counsellor*. (26~~5~~-35~~4~~ year-old cis-man. Baseline testing, false negative result)

Both participants reported low HIVST acceptability following their experiences, were suspicious of the technology and reluctant to endorse self-testing to their peers.

> *No, at first, before the testing I was promoting it to my friends, they were a bit sceptical about it because they were of the same opinion as me, self-testing doesn't sound very accurate, so they weren't taking it on board, so to speak. But after what happened with me being diagnosed positive for HIV, I never promoted self-testing again.* (2~~5~~6-35~~4~~ year-old cis-man. Baseline testing, false negative result)
>
> *Interviewer: Do you think that you would use self-testing again if it was available to you?*
>
> *Participant: Do you know I would do, but I would want someone there with me to walk through the process, but self-testing at home on my own?* […] *I would be very wary of it* […] (2~~5~~6-35~~4~~ year-old cis-man. Baseline testing, false positive result)

Intervention harms

Harms related to how the intervention functioned were reported by two individuals. Accounts focused on feelings of guilt and shame when they completed online risk surveys, and (for one), the cyclical nature of the repeat testing intervention. This component of the trial was designed as part of the intervention and intended to prompt reflection about past risk.

> *Once you've entered into the trial and then it's like, so, why did you feel the need* [to test]*? What sort of person do you think you have become where you feel that you have to be tested? And it also made me think, why do I feel the need to be tested so regularly at the normal clinic?* […] *Am I living a normal sort of lifestyle? Is my lifestyle, is it out of control? Is it the sort of lifestyle that I want? And how, at my age, did I get myself into this position?* (45~~60~~+ year-old cis-man. Repeat testing, well-being harm)
>
> *I don't know, in an odd way kind of makes you think about it more, the fact that it's going to come. Whereas you know, like if you just think oh I've just been and done something risky then I should got to a clinic. It's not like it's on your mind every...and I guess it's the taking part because it's like where is it going to be delivered to in two months' time, but I suppose*

> *they're not necessarily things that I can separate*. (18-25~~4~~ year-old cis-man. Repeat testing, well-being harm)

For both, narratives describing their experiences focused on their internal monologue and feelings about their circumstances. For one this was living on his own and for the other it was related to not being open about his sexual orientation with his family. These narratives were focussed on guilt, shame and loss of control. Although the surveys triggered these feelings, using HIVST kits in the home also increased this because of the incursion of healthcare into the private sphere, an issue specific to HIVST:

> *I guess because it's quite a clinical thing actually, you know, when you think about it. It's quite a clinical thing to be doing in your own room. It's like, you know, something you would ordinarily have done by someone who's trained, but you're having it in a different way and you're having it in a completely different setting. So maybe it would be easier to, kind of, make it be I guess more normal in that clinical…* [setting] (18-2~~4~~5 year-old cis-man. Repeat testing, well-being harm)

For both participants, acceptability of HIVST was still generally high: self-testing was felt to be a useful intervention which met their testing needs, it was primarily the psychosocial components and cyclical nature of the intervention which led to well-being issues.

> *I think so* [would use HIVST again]. *I mean, I guess if I wasn't living with my parents and I could just order the kit, then it would come, rather than not being every three months then I think I would have much of a problem with that, I think, but it's just all those other elements that kind of add up, I think.* (18-2~~4~~5 year-old cis-man. Repeat testing, well-being harm)

> *If it was a limited amount of self-testing available then I would say I probably wouldn't have used it, because going to the sexual health clinic doesn't faze me any more. And if there wasn't enough to go round, then I would say, well, let somebody else have it. The fact that I can get tested at home is great. But the fact that it no longer fazes me to go to a sexual health clinic and get tested, as I say, it's possibly for somebody else. But getting tested for HIV at home is great. Given the opportunity, I would recommend it to anybody.* (4~~6~~5~~0~~+ year-old cis-man. Repeat testing, well-being harm)

Socially emergent harms

Four participants reported harms described as socially emergent in that they arose from the social context of their lives. For one, this was a negative impact on a relationship, while for three this was pressure to test for HIV when they did not want to.

One participant reported a difficult relationship with his partner which was characterised by jealousy and suspicion. His partner's discovery of his HIVST led to substantial discord and contributed to eventual relationship breakdown. His narrative, however, described the inevitability of the outcome: had the discovery of the kit not provoked breakdown another event would have precipitated it.

> *He always had, like, anxiety issues and, like, intimacy issues and jealousy issues*. […] *He was very worried about the attention I get on nights out and stuff like that. And most of the time*

> *it would just be friends or people I know from, like, years and years and years ago. So, he was always very concerned, like, you know, you're chatting to that person, what you chatting about, what are you planning on doing, are you going to go out with that person and stuff like that*. (26-35 year-old cis-man. Repeat testing, relationship harm).

Self-testing acceptability was no diminished by the relationship breakdown and the participant continued to recommend HIVST to others.

> *For me, I think it's* [HIVST] *a brilliant thing. I mean when I was on the trial, I was genuinely telling people,' yeah I'm doing this trial and it's really good, you should do it.' I was, like, actually, trying to get people to sign up for it.* […] *But, no, I think that the whole self-testing thing is so much easier and so much more…it puts your mind at ease more. Because, again, you're at home, you can have your own, sort of, network here if you want it, or you can do it on your own, then, like, if you do test positive you can cope with that on your own and then deal with it, like, in a more professional capacity, and it's just easier.* (26-35 year-old cis-man. Repeat testing, relationship harm).

Pressure or persuasion to test during SELPHI was experienced by three individuals in different circumstances: for one it came from a friend concerned for his health. For another pressure came from a partner in response to his own worsening mental health due to HIV anxiety. The final participant described being forced to test in a clinic by the police after being the victim of a violent sexual assault when he would have preferred to use HIVST; his experience was thus not related to HIVST or his participation in SELPHI.

Both individuals pressured to test by those in their social networks described ambivalence and anxiety around testing for HIV while also recognising their own unmet testing need. For one this unmet need was the source of significant stress and negatively impacted their well-being.

> *It was an ex-partner. I had said to them, obviously, I was over-thinking at the time and they basically said to me 'the only way that you're going to get rid of that feeling is by just going for a test, rather than just pushing it to the back of your mind'. Because they were like, 'imagine if you did have it and you didn't know and then you found out at a later stage'. And that's what pressured me into going for it because I think it made me worry more and it actually made it more of a priority to go for a test which they shouldn't have made me worry even more* […] (18-25 year-old cis-man. Repeat testing, pressured to test).~~(25-34 year-old cis-man. Baseline testing, pressured to test).~~

Although their narratives both described significant ambivalence around the experience, they were happy they had tested in retrospect and both felt more confident with future testing, and reported high HIVST acceptability:

> *It* [testing] *just seems so much more reachable. I feel comfortable doing it now. And I think, more than anything, it's put my mind at rest that it is getting easier to be able to be tested for this* […] (18-25 year-old cis-man. Baseline testing, pressured to test)

> *I think it made me feel like, now, I think if I go and get the test done it's a big relief for me because at least I'll end up finding out what the outcome is, rather than just thinking, oh, will it or won't it? I think it just gets the hard... Going for the test, just gets the hard bit out the*

way and it does help to relieve some worry. (18-25 year-old cis-man. Repeat testing, pressured to test) (25-34 year-old cis-man. Baseline testing, pressured to test).

Interviewer: *And in terms of the pressure that your friend put on you to test, do you think that's in any way shaped how you feel about testing?*

Participant: *Yes. I think it's made it... It's made me more aware of it, for sure. And it's made me keep on top of it. And I feel that it's... It's not that I didn't think it was important before, but I thought it's something that I'm never going to have to do. But now it feels like it's definitely something that everybody should be doing. So it just feels more normalised now, which is good. But, yes.* (18-25 year-old cis-man. Baseline testing, pressured to test) (25-34 year-old cis-man. Baseline testing, pressured to test).

For participants who were pressured to test when they did not want to, HIVST through SELPHI was simply the most accessible test available to them at that time. If HIVST was not available this pressure likely would have led them to test using another modality.
